# Smarter Than Thou, Holier Than Thou: The Dynamic Interplay Between Cognitive and Moral Enhancement

**DOI:** 10.3389/fphar.2018.01189

**Published:** 2018-10-29

**Authors:** Gabriela Pavarini, Alex McKeown, Ilina Singh

**Affiliations:** Department of Psychiatry, Wellcome Centre for Ethics and Humanities, University of Oxford, Oxford, United Kingdom

**Keywords:** moral enhancement, cognitive enhancement, smart drugs, moral bioenhancement, neuroethics, empathy, cognition, morality

## Abstract

The debate about the desirability of using drugs to enhance human skills encompasses cognitive abilities such as memory and attention, and moral capacities such as emotional empathy and a sense of fairness. These two strands of literature in bioethics have grown relatively independent from each other, and an implicit framing assumption has emerged suggesting that apparently morally neutral cognitive capacities and paradigmatically moral capacities are distinct and vary independently of each other. Here, we identify key distinctions between competing accounts of cognitive enhancement and moral enhancement and argue that, despite the polarized nature of the bioethical debate, cognitive and moral capacities are intertwined. For example, moral behavior can be improved by enhancing “morally neutral” abilities such as attention span; and cognitive skills can be honed by means of socio-moral interaction. Further, cognitive skill is frequently assigned the abstract status of virtue and treated in the same way as more paradigmatically “moral” traits. We argue that the distinction between moral and cognitive enhancement is more apparent than real, since despite being nominally treated as distinct, cognitive and moral skills are frequently interdependent. As such we present evidence to support the claim that the enhancement of these two kinds of capacities cannot be clearly disaggregated from each other in the way that the theoretical poles of the debate in the literature suggest. We synthesize relevant scientific and bioethical literature and combine it with a line of analysis derived from Peter Hacker to show more clearly the terms of what can be said intelligibly about cognitive and moral skills and their enhancement. As a result of this analysis, we conclude that ethical questions in human bioenhancement are only fully intelligible at the level of persons imbued with feelings, thoughts, intentions, desires, values, and abilities, embedded within a particular social context, rather than at the level of pharmacological modulation of particular cognitive or affective capacities which, though conceptually distinguishable, in the embodied context of moral agency are profoundly intertwined.

## Introduction

Cognitive enhancers are normally defined as drugs or other biological methods aimed at boosting cognitive capacities, including mental energy, attention, working memory, wakefulness, or task-orientated motivation (Bostrom and Roache, 2008; Bostrom and Sandberg, 2009). Moral enhancers as understood in the literature are pharmacological or other biological methods of improving people’s ethical judgment and moral behavior, where improvements in this context are typically associated with prosociality (Douglas, 2008; Savulescu and Persson, 2012). Different arguments have been raised for and against these two apparently different types of enhancement, and these two strands of literature in bioethics have grown relatively independent from each other. An implicit framing assumption has also emerged which suggests that cognitive and moral skills are distinct and vary independently of each other. For example, it has been suggested that the enhancement of cognitive capacity should be accompanied by enhancement of moral capacity, since for these to become misaligned with each other would bring about harmful outcomes, such as in the development and use of weapons of mass destruction (Persson and Savulescu, 2008).

This paper scrutinizes the intersection of the enhancement of apparently morally neutral cognitive skills, such as attention, and apparently paradigmatic moral traits, such as empathy and a sense of fairness. We argue that the distinction between moral and cognitive enhancement is more apparent than real and present evidence to support the claim that the enhancement of these two kinds of capacities cannot be clearly disaggregated from each other in the way that the theoretical poles of the debate in the literature suggest.

To investigate this network of issues, we first outline ways in which bioethical accounts of cognitive enhancement differ from accounts of moral enhancement. We then examine relevant empirical data from psychology to understand how cognitive and moral skills are intertwined and how they resist in a real-world context the theoretical distinction advanced within bioethical analysis. Finally, we will use these analyses to show how a linguistically informed approach drawing on Peter Hacker’s critique of neuroscience can clarify what can be said intelligibly about cognitive and moral skills and their enhancement.

## The Ethics of Enhancing Skills and Virtues: Parallels and Contrasts

### Enhancing for Oneself, Enhancing for Others

Several parallels can be drawn between implicit assumptions in bioethical accounts of cognitive enhancement and moral enhancement. First, assumptions of interest seem to differ: whereas cognitive enhancers to boost performance are typically assumed to appeal to cognitively normal, healthy individuals, the appeal of moral enhancers is often framed in terms of the benefits available to people whose behavior is considered divergent from social norms and causes negative consequences for self and/or others (the complexities of defining “a morally normal agent” notwithstanding^1^). Although these appear to map on to standard categories of therapy and enhancement and the difference between them, namely that the first corrects an abnormal state and the second does not, both may still be said to enhance insofar as they confer an improvement irrespective of the baseline at which the intervention is applied (Scully and Rehmann-Sutter, 2001).

Underlining the probable desirability of cognitive enhancement, Chatterjee (2004) holds that the arrival and use of effective drugs are inevitable given the kinds of things that modern humans tend to value, and Dubljevic (2012) advocates the need for strategies for managing and limiting (in particular, unequal) access to relevant enhancement products. By contrast, Bronstein (2010, p. 86) contends that bio-engineered virtue “seems unlikely to find a willing public” and Tonkens (2014) claims that parents are unlikely to express a desire to morally enhance their children. As noted above, the term moral enhancement has been used to refer to interventions aimed at “deficiencies” in morality rather than increasing the moral qualities of the average person. For example, the use of “moral enhancers” in the justice system, such as chemical castration of sex offenders, has received considerable attention in bioethical debate (Douglas, 2008; DeGrazia, 2014; but see Horstkötter et al., 2012 for an objection to using the term in forensic contexts). By contrast, bioethical accounts of cognitive enhancement mostly focus on improving functioning in individuals without a cognitive impairment (Bostrom and Sandberg, 2009).

A second implicit assumption found in the bioethical discourse surrounding cognitive (but not moral) enhancement is that cognitive enhancers will be used for achieving self-interested goals or “in order to gain advantage over others” (Dubljevic and Ryan, 2015, p. 30), thus raising concerns about fairness. For example, Goodman (2010) suggests that the use of cognitive enhancers is acceptable as long as doing so does not produce a zero sum outcome, that is, where the consequence of person A using an enhancement is that person B loses out. Cakic (2009) makes a related argument framed in terms of cheating, and holds that cognitive enhancement is acceptable as long as doing so does not cheat someone else out of something that they deserve. Similarly, Sahakian and Morein-Zamir (2011, p. 202) state that: “rather than advocate for overall inclusion or exclusion of PCE [pharmacological cognitive enhancement] use in healthy individuals…[we] would encourage their responsible use” and emphasize the principle that PCE should not cause harm or exacerbate social problems such as inequality. Related concerns surrounding social justice have also been raised by a number of other theorists (Kass, 2003; Kazin, 2004; Sandel, 2004; Schermer, 2008; Ray, 2016).

These themes appear salient in and reflected by public intuitions about cognitive enhancement. Empirical research on public attitudes toward cognitive enhancement has pointed to feelings of ambivalence and discomfort (Bergström and Lynöe, 2008; Racine and Forlini, 2010; Singh et al., 2014; Dijkstra and Schuijff, 2016; Bard et al., 2018) and a general concern that it may produce effortless, undeserved, or hollow achievements (Sabini and Monterosso, 2005; Fitz et al., 2014; Faber et al., 2016). Some theorists have also highlighted these feelings and their significance, suggesting that some people feel a persistent “angst” in relation to cognitive enhancement (Reiner, 2013), and that others are threatened by it from a competitive standpoint (Greely, 2010).

Analogous concerns surrounding unfair advantage, social justice, and responsible personal use are not commonly raised in the context of moral enhancement, however. The justification for biomedical moral enhancement turns on it being likely to make individuals more other-considering and prosocial, which is presumed to be beneficial to others (Douglas, 2008). Debates rarely discuss *individual* benefits that may arise from the use of moral enhancers, and the possibility that users might seek these drugs to achieve personal purposes, including recreational or self-improvement goals. Indeed, one argument *against* the wisdom of moral bioenhancement is that it would undermine individual freedom (Harris, 2011; Simkulet, 2012); and would lead to the prioritization of contestable societal values to the detriment of individual interests (Rakić, 2017). As a corollary, zero-sum concerns and accusations that the user would be undeserving of any “gains” the enhancer might provide, are typically absent from the moral enhancement literature (Douglas, 2008, 2013; Crockett et al., 2010; Savulescu and Persson, 2012). The discussion is instead framed in terms of intentionality, and centers on whether a moral act that results from an enhancer can be described as genuine and deliberate (Chan and Harris, 2011; Rakić, 2017).

Taken together, the accounts presented so far reveal a contrast between intuitions surrounding these different types of enhancement, with cognitive enhancement being primarily thought of as promoting individualistic goals (with the agent largely in control of deciding whether to take a cognitive enhancer), and moral enhancement as advancing collective values (with the agent more likely to be encouraged by others to take a moral enhancer, for one reason or another).

However, other bioethicists do not draw such a clear distinction between cognitive and moral enhancement; indeed, some highlight potential collective benefits that cognitive enhancement might confer. For example, Bostrom and Sandberg (2009, p. 330) write:

At a societal level, the consequences of many small individual enhancements may be profound. A relatively small upward shift of the distribution of intellectual abilities would substantially reduce the incidence of retardation and learning problems. Such a shift would likely also have important effects on technology, economy, and culture arising from improved performance among high IQ groups.

In line with this view, others suggest cognitive enhancers can promote social goods if they are used by particular groups in society, for example by judges (Chandler and Dodek, 2016), surgeons (Vincent, 2011; Goold and Maslen, 2014), or pilots (Santoni De Sio et al., 2014). Similarly Harris (2013) suggests that cognitive enhancement that promotes rational thinking and careful calculation of consequence can help individuals act in ways that are in fact *consistent with* collective values. Finally, some endorse cognitive enhancement as a means to promote fairness (Savulescu, 2006; Bostrom and Roache, 2008; Ray, 2016), if provided to those at the lower end of the normal distribution range for particular cognitive capacities. Savulescu (2006, p. 334) holds that we should promote those things which will maximize the good, one of which is better cognition for as many people as possible:

…what IQ is necessary for a decent chance of a decent life? Perhaps, in a technologically sophisticated society, people would significantly benefit from a higher IQ. An IQ of 120 is needed to be able to complete tertiary education. Justice/Fairness requires we get as many people as possible up to the minimum IQ necessary for a decent chance of a decent life. Fairness thus requires enhancement. Far from being opposed to enhancement, justice requires enhancement. It is on these grounds that we choose to treat those currently with an IQ less than 70. But where we set the minimum threshold for treatment or enhancement is up to us.

Crucially, these views that emphasize the “collectivist” potential of cognitive enhancement are not commonplace or typical of those presented in the media, or expressed by healthcare providers or the public, which, much as with the literature that is suspicious of cognitive enhancement, instead tend to focus on the elective use of cognitive enhancers as a mean to promote individual, rather than societal, interests, and achievement (Schelle et al., 2014).

### Defining How to Enhance

As noted earlier, the moral enhancement literature is characterized by an ongoing debate about the extent to which moral behavior emerges from deliberative, cognitive processes or by affective responses. The debate took the form that it assumes today via a series of disagreements that began around a decade ago between, on one side, Tom Douglas, Julian Savulescu, Molly Crockett, and others, and John Harris in a relative minority on the opposing side. For example, Douglas (2013, p. 162) holds that “The distinctive feature of emotional moral enhancement is that, once the enhancement has been initiated, there is no further need for cognition: emotions are modified directly.” By contrast, Harris (2013, p. 116) argues that since morality is a matter of all-things-considered rational judgement, it cannot be confected by making people feel a certain way:

…no one who claims to be acting morally or out of moral conviction or principle can resist accountability for what they claim to believe or do in the name of morality. And this means they must always be prepared to offer a reasoned defense and justification of their morality or elements of it. It would never be enough or indeed even respectable for the reply to be “I just felt like it.”

Harris (Ibid.) goes on to emphasize this by citing instances in which it might be morally obligatory for us to deliberately hurt people, either in self-defense or in defense of others, even though we might not wish to do so. In these instances, the all-things-considered best course of action may be one that one is reluctant or indisposed to pursue, and, he argues, in making us more empathetic the upregulation of prosocial neurotransmitters may make us less likely to meet what are, in fact, our moral obligations, however unpalatable they may be.

According to this line of argument, therefore, to draw an equivalence between prosociality and morality is a significant assumption in need of justification (and, indeed, an assumption with which he disagrees). From here, advocates of moral bioenhancement employ various forms of the generic argument that, since moral behavior is at least *in part* affective, modulating people’s emotions such that they are more likely to treat each other more kindly, empathetically, and altruistically will be at least in part constitutive of moral enhancement.

The crux of the problem then, as laid out in the bioethics literature on moral enhancement, is whether moral enhancement is a function of emotional manipulation alone, or a function of the manipulation of both reason and emotions. As with definitional debates in bioethics in general, and indeed in philosophy more broadly still, the kernel of the argument laid out here has been batted back and forth by proponents of the opposing views, without any clear resolution. Concurrently, numerous contributions have been made to the debate by others seeking to reconcile or synthesize the two accounts, with some success. For example, it has been disputed that either affective or cognitive aspects of morality take priority over the other, since both are involved in moral decision-making (Baertschi, 2014); indeed Jotterand and Levin (2017, p. 5) have labeled a putative opposition between reason and emotions as a “false dichotomy.”^2^

In sum, the role of emotions is an important topic of discussion in the moral enhancement literature. However, in the cognitive enhancement literature, there is no comprehensive discussion on the role of emotions in the promotion of cognitive performance, with a few notable exceptions. For example, Kjærsgaard (2015) recently pointed out that motivational components may play an important role on the performance enhancing effects of smart drugs. This suggestion was based on studies in which users described the effects of smart drugs as at least partially affective or motivational, pointing out to an increase in drive and interest for the subject matter (DeSantis et al., 2010; Ilieva and Farah, 2013; Vrecko, 2013). Similarly, a randomized controlled trial suggested increased task motivation in healthy participants who had received modafinil (vs. placebo) (Müller et al., 2013). Ethical accounts of motivational enhancement in the context of cognitive enhancement are scant; however, in a study investigating lay people’s opinions on motivational enhancement, Faber et al. (2015) found that participants considered cognitive enhancers that act primarily on motivation to be less wrong than those that act on cognitive skills.

On a cautionary note, it is important to point out that the putative enhancing effects of smart drugs on both motivation and cognition are still under dispute (Farah, 2015). A throughout discussion of the effectiveness of cognitive and moral enhancement methods is beyond the scope of this paper. Instead, we focus on drawing parallels between implicit assumptions in bioethical accounts of cognitive enhancement and moral enhancement, without claiming that these methods are effective or in what way.

With regards to the role of emotions *versus reason* in moral enhancement, some theorists have abstained from making a strong commitment to either side of the debate, by adopting a more skeptical position. Shook (2012, 2016) argues that our understanding of the biological basis of decision-making is still too primitive and limited for us to be able to say with accuracy that moral behavior can be reliably enhanced using pharmacological agents. Sparrow (2014) takes a pragmatic approach that favors attention to social determinants of (moral) behavior, about which much is known, over the relatively thin evidence for biological determinants. Thus Sparrow argues that social and political change rather than pharmaceuticals are required to improve moral behavior. This leads him to a wider focus on social justice over a narrow focus on moral enhancement as a public good:

When it comes to thinking about the implementation of any real-world program of moral enhancement, then, the political issues over-determine the ethical questions. Without an educated, empowered, and rights-respecting citizenry, moral enhancement will be too dangerous to attempt. With such a citizenry, it will most likely be unnecessary. The urgent imperative in the current moment is not moral enhancement but social justice — the pursuit of which is perhaps less novel and is certainly less headline grabbing than “moral bioenhancement” but is much more likely to address the problems that Savulescu and Persson profess to be concerned about. (Sparrow, 2014, p. 30)

Consistent with this type of critique, others hold that the kinds of arguments made by the main proponents of moral enhancement are merely reflective of the norms of their social milieu, as discussed in the following section.

### Putting Enhancement Into Context

As well as a degree of complexity with regard to what counts as moral enhancement, the apparently intractable debate on the role of affect versus reason on moral enhancement also reflects the complexity of defining what is *worth enhancing* from a moral standpoint. There is a clear acknowledgment in the moral enhancement literature of the cultural plurality of moral norms (Bublitz, 2016), and that people will hold different views about what should be the targets of moral enhancement, varying from obedience to social rules, respect to authority and loyalty to compassion, openness, and audaciousness. Analyses from across the moral bioenhancement literature flag up the social context in which one must act as indispensable determinant of whether one’s actions can be properly judged as moral or not (Dees, 2011; Wiseman, 2014; Focquaert and Schermer, 2015).

Much less debate seems to exist with regard to what constitutes cognitive enhancement. However, some writers in the bioethics literature have questioned the intrinsic value of better cognition and the universal desirability of enhancing cognitive skills. Capps et al. (2012, p. 263) argue that in prizing and prioritizing as it does the importance and value for individuals of high performance cognition, the pro-enhancement case is implicitly libertarian and as such inherently ethically problematic from the point of view of social justice. The social milieu in which these arguments are made and by whom is adduced as evidence for why cognitive enhancement is unethical:

when a libertarian describes a technology as an “enhancement”, they are indicating its use in unique and partial social circumstances, and therefore, they cannot possibly be claiming, with any ethical authority, that its legitimate use can be universalized to other contexts. When proponents of enhancement are in agreement, they are making a statement to the effect that technology is to be accepted on the basis of a particular conception of how it is intrinsically linked to “progress”. This is merely the perpetuation of a narrow set of “legitimate choices” that are compatible with one privileged class’s idea of “the good life”.

The authors thus argue that a neutral or positive attitude toward enhancing cognition reflects a view of life held by a privileged class which is not sufficiently other-considering and assumes that because cognitive enhancement would be good for them it must necessarily be good for all. Others add that most of the bioethical debate has been led by relatively privileged western individuals and as such they attribute this as a reason why the enhancement debate in general has assumed an individualistic, neoliberal flavor, and remind us that other, potentially more pluralistic and community-minded, perspectives on the issues at stake are possible (Buchanan, 2008; Bradshaw and Ter Meulen, 2010; Serna, 2012). By extension, all these accounts implicate contextual factors as vital for any comprehensive definition of cognitive enhancement.

However, the discussion tends to concentrate on whether the enhancement of cognition, as a unitary, commonly understood concept is in itself valued by many or a few. Less effort is dedicated to discussing the plurality of cognitive skills and their varying normative value across cultures, including different ways of perceiving objects, paying attention, reasoning, and so on.

## The Separability and Interplay Between Moral and Cognitive Enhancement

In the previous sections, we outlined ways in which bioethical accounts of cognitive enhancement differ from accounts of moral enhancement. We have identified a few distinctive features, including: (1) an overall bias toward representing cognitive enhancement as promoting individualistic goals, and moral enhancement as promoting collective goals; (2) a focus on use by healthy users in the former case, rather than on clinical applications in the latter; (3) a focus in the cognitive enhancement case on threats to fairness and social justice that is largely absent in the moral enhancement debate; (4) a focus on the role that emotional states play in moral enhancement, which is not as prominent in the case of cognitive enhancement; (5) a greater consensus within the cognitive enhancement literature than within the moral enhancement literature about what “enhancement” consists of and what is worth enhancing.

Some of the distinctive features found in the bioethical accounts of moral as compared to cognitive enhancement are consistent with empirical studies that investigate the ways in which moral virtues and cognitive skills structure self- and other-perceptions. For example, information about morality is more often sought when perceiving or judging other people, whereas information about cognitive skill plays a more central role in self-judgments (Wojciszke, 2005; Abele and Wojciszke, 2007). Other studies have shown that self-ascribed cognitive skills, such as intelligence, predict self-esteem more strongly than self-ascribed virtues such as kindness or fairness (Wojciszke et al., 2011; Wojciszke and Sobiczewska, 2013). By contrast, there is evidence that, when judging other people, negative information about morality has primacy over information about competence, and is weighted more heavily when forming a global evaluative judgment of that person (Wojciszke, 2005). Finally, neuroscientific studies suggest that watching others’ virtues and skills recruit partially dissociable brain areas and distinct eye gazing patterns (Immordino-Yang et al., 2009; Yang et al., 2018).

However, neither the distinctions that we are inclined to make nor the polarized nature of the bioethical debate imply that cognitive and moral capacities are structurally independent of each other, or that they can be clearly disaggregated from each other in practice. Despite the strong psychological bias towards treating these as separate dimensions, we contend that the mechanisms underlying the expression of virtue are closely intertwined with those underlying the expression of cognitive skill.

The synthesis we present adds to previous bioethical attempts to reconcile cognitive and moral enhancement, such as papers that identify collective benefits of cognitive enhancers (Bostrom and Sandberg, 2009; Goold and Maslen, 2014; Chandler and Dodek, 2016). The novelty of our contribution lies in pointing to a more direct interplay between cognitive and moral capacities. In the following sub-sections, we present a synthesis of evidence indicating that (a) cognitive enhancement can lead to improvements in moral capacities, and that (b) moral enhancement can lead to improvements in cognitive capacities. We also discuss evidence suggesting that certain cognitive capacities are moralized and treated in the same way as more paradigmatic virtues such empathy or altruism, thereby blurring distinctions between these two types of enhancement.

### The Effect of Cognitive Processes on Moral Enhancement

Implicit across much of the cognitive enhancement literature is the general notion that morally neutral cognitive skills such as attention or working memory can be selectively enhanced without an impact on one’s sense of morality. In other words, the position advanced is that cognitive enhancers selectively act on so called “amoral traits,” without affecting a person’s moral capacity as such, assuming for now that by “moral” we mean something more obviously prosocial than these traits. However, there is empirical evidence to support the idea that improvements in morally neutral traits such as attention management, thought suppression and wakefulness also play a key role in conforming to standards of right behavior and exhibiting traits typically considered to be morally virtuous. This implies that efforts to selectively improve morally neutral cognitive skills may have an impact on people’s moral thinking and behavior.

For example, there is evidence that wakefulness, a trait that normally falls outside the realm of *moral* consideration, powerfully affects moral behavior. Across a series of studies, sleep deprived participants were more likely to show workplace deviance (Christian and Ellis, 2011), negative implicit attitudes toward social groups (Alkozei et al., 2017), cyber-loafing (Wagner et al., 2012), and cheating behavior in a trivia test (Barnes et al., 2011), compared to well-rested counterparts. Unethical behavior was also shown to be more frequent in the afternoon than in the morning, when individuals are normally better rested (Kouchaki and Smith, 2014).

Similarly, cognitive control seems to be functionally linked to morally relevant behavior (Baumeister and Exline, 1999; Muraven et al., 2006; Gino et al., 2011; Osgood and Muraven, 2015). In a key experiment (Mead et al., 2009), participants played an economic game after completing either congruent or incongruent trials of a Stroop task. This is a standard reading task where participants read words that refer to colors, written either in matching colors (for example, the word “green” written in green), or mismatching colors (for example the word “green” written in red). Mismatching or incongruent trials are expected to engage selective attention skills and deplete one’s “self-control muscle.” Even though the Stroop task is not thought of as having inherent moral value, participants who had completed incongruent trials before were more likely to cheat on the subsequent economic game.

Conceptual replications further suggested that participants who had their cognitive control skills depleted were less charitable (Xu et al., 2012) and less likely to help victims of a tragedy (DeWall et al., 2008). These effects were not mediated by emotional states, for example, frustration (DeWall et al., 2008). The opposite also seemed to hold: participants who practiced techniques to focus and calm the mind without any reference to moral content were more likely to offer their seat to a suffering stranger in a waiting room in comparison to participants who had not undergone such training (Condon et al., 2013).

It is possible that these effects are due to a general effect of cognitive control on people’s ability to forego immediate gratification for a greater later reward, an ability that has been shown to predict helpful and cooperative behavior (Mischel et al., 1989; Shoda and Mischel, 1990). However, it is worth mentioning that despite neurological evidence suggesting otherwise (Peters and Büchel, 2011) a recent behavioral study failed to find a direct relationship between participants’ tendency to avoid distracting information when completing a task (*cognitive control*) and delayed gratification (Scherbaum et al., 2018). Therefore, future research should clarify the link between cognitive control and self-control, as well as the process whereby cognitive control might facilitate morally relevant behavior.

It is also important to point out that we do not claim here that cognitive enhancement *necessarily* leads to parallel improvements in moral behavior. However, the findings discussed in this section illustrate the interplay and potential overlap between general cognitive capacities that are often targeted by cognitive enhancement efforts and the psychological architecture that supports behavior typically associated with “being moral.” This relationship is also visible when viewed in the opposite direction, as we discuss next.

### The Effect of Moral Processes on Cognitive Enhancement

Accumulating evidence suggests that the state of one’s socio-moral relationships has pervasive effects on cognitive performance. Among pre-schoolers, solitary play (and for boys, also antisocial behavior) was shown to be correlated with difficulties with emergent literacy (Doctoroff et al., 2006). On the other hand, greater frequency of prosocial behavior in early childhood, including helping, sharing, cooperating, and comforting, strongly predicted better academic achievement in adolescence. Early academic performance did not account for any variance in teenagers’ achievement when controlling for differences in early prosociality (Caprara et al., 2000). Similarly, from kindergarten to high school, emotional learning programs designed to foster empathy and positive social connections were found to improve academic performance in comparison to control programs (Denham and Brown, 2010; Durlak et al., 2011). The affective qualities of teacher–student relationships have also been shown to affect both engagement and achievement at school (Roorda et al., 2011). Studies with adult population have revealed similar findings. For example, employees who reported having friends at work were shown to be more productive than those who did not (Rath, 2006). Finally, in a representative sample of US elderly participants, those who had the highest levels of social integration had a memory decline twice as slow as those with the lowest levels of low social integration (Ertel et al., 2008).

Experimental laboratory evidence further supports a link between socio-moral processes and morally neutral cognitive skills or individual achievement. Participants who had been asked to recall a time they felt the moral feeling of gratitude were almost twice more likely to display self-control in a subsequent delayed gratification task, in comparison to control participants who had been led to feel happy or neutral (Dickens and DeSteno, 2016). In another study, participants who were subject to a brief experience of social exclusion, in this case reading a message saying they would likely end up alone in life, demonstrated significant reductions in certain cognitive capacities including logical reasoning, as measured by a Graduate Record Examination test, and a standard IQ test (Baumeister et al., 2002). Even though social exclusion cannot be equated to moral de-enhancement, these findings support our argument that socio-moral functioning is intertwined with cognitive functioning.

The notion that improved moral competency facilitates the acquisition of putatively cognitive skills is consistent with the more general notion that cognition is a social and collaborative process, as put forward by a number of developmental psychologists (Bronfenbrenner, 1979; Vygotsky, 1980; Rogoff, 1998, 2003). Cognitive development does not occur in a vacuum; it depends greatly on social engagement, communication, and shared participation in socio-cultural practices, and promotes shared goals. In other words, processes that might be thought of as purely individual such as remembering, planning, thinking, and reasoning are in fact closely tied to social goals and an individual’s functioning as a member of a moral community.

We should be clear that it is important not to oversimplify and misrepresent the relation between cognitive and moral capacities. We have all encountered cases where exceptionally gifted individuals in the cognitive domain were found guilty of seriously immoral behavior or cases where highly moral people seemed endowed with only modest cognitive ability. We do not aim to claim that cognitive and moral capacities influence each other in ways that are straightforward or uniform. However, the evidence that we adduce supports our claim that the two kinds of capacities should be understood as nevertheless existing in a dynamic and interdependent relationship that casts doubt on the putative separability between them that is suggested by the relevant literature regarding their enhancement.

### Cognitive Skill as a Moral Norm

It is uncontroversial to point out that there is a vast and culturally, historically, societally, and geographically conditioned plurality of moral norms. As such there is commensurately wide variation of views about what traits are desirable and worthy of enhancement, and indeed what counts as a “moral” trait, rather than an “immoral” one in the first instance. Despite the apparent distance between cognitive and moral capacities, we contend that there is also variation in terms of what kinds of putatively cognitive skills are valuable and, thus, presumably would be desirable targets for enhancement. Although it might appear that certain capacities, for example focused attention or alertness, have no particular evaluative dimension, such capacities are valuable, or not, in relation to their context and the norms to which individuals in that context conform. In other words, what is considered cognitively good, skillful, and a worthy target of enhancement depends on culturally shared norms. Indeed, the scale at which norms can be shared is considerable. Certain kinds of cognitive skills, for example, analytical thinking or mathematical ability, are in high demand across highly developed societies in view of their usefulness relative to one’s success in such societies. This attribution of value is often implicit; however, in some cases, cognitive capacities are explicitly understood as *moral* values, both in individual lives and at the level of culture. In other words, certain cognitive capacities are awarded the status of a virtue, since they are evaluated with respect to the desirability of a particular goal, and tacitly ranked according to their value in the same way as traits or capacities that we might view as more paradigmatically “moral” such as obedience or kindness.

Across various cultures and historical periods, different cognitive strengths involving knowledge acquisition and use have been placed in the domain of virtue, including traits such as critical judgment, open-mindedness, perspective, curiosity, and creativity (Peterson and Seligman, 2004; Dahlsgaard et al., 2005). There is also evidence that reliance on reason and evidence in the formation of belief is considered by some as a constituent of moral capacity. According to these accounts rationality is treated as a normative ideal; as such rational thinking in these cases is considered prototypical of a moral role model, whereas irrationality elicits negative moral emotions and a desire for punishment (Ståhl et al., 2016). Finally, in Aristotelian philosophy, practical wisdom, which involves cognitive processes of deliberation and discernment, has been referred to as not only a virtue, but a *master* virtue that can give rise to all others (Aristotle, 1999; also see Schwartz and Sharpe, 2006 for a modern psychological reinterpretation).

Findings such as these seem to suggest that human societies often tend to value cognitive advancement in its varying forms, and implicitly or explicitly imbue different skills with the status of valuable or virtuous. It is not obvious to us that this attribution of value is fundamentally different from attributions of value to paradigmatically “moral” traits such as loyalty and generosity. When the line between what constitutes valuable expressions of morality and cognitive skill is blurred, there might be no reason to think that normative decisions with regards to enhancement in the former case should differ from the latter.

In the following discussion, we strengthen our central claim that cognitive and moral skills and capacities are integrated and interdependent, rather than clearly separable. We draw on Peter Hacker’s critique of neuroscience to argue that these skills and capacities can only be fully understood as representations of a *person*, and cannot be sensibly comprehended in abstraction at either the micro scale at the level of discrete cognitive or affective processes, nor without taking into account the particular social context in which the *person* in question must think, feel, judge, and act.

## Speaking Intelligibly About Cognitive and Moral Skills and Their Enhancement

So far we have attempted to illustrate the interplay between, on one hand, capacities conventionally considered morally neutral such as wakefulness and cognitive control, and on the other hand, apparently paradigmatically moral traits such as compassion and helpfulness. We have presented evidence to support the claims that: (1) enhancing cognitive skills often considered morally neutral such as executive functioning can lead to enhancements in behavior typically associated with being “good” or “bad”; (2) capacities that we conventionally define as “moral” such as prosociality and empathy can hone the development of apparently morally neutral cognitive skills; and (3) certain cognitive capacities are awarded the status of a virtue, and ascribed moral value in the same way as more paradigmatically “moral” skills.

It is evident from empirical data and from the inconclusiveness of the bioethical debate that accurately identifying the constituents and determinants of cognitively and morally skilled behavior is complex and does not admit of unitary explanations. For the reasons outlined, the complexity partially arises from conflicting accounts of what is considered good for individuals and societies. Norms regarding the desirability of certain capabilities or actions are socially conditioned and cognitive skills may or may not be deemed as constitutive of those norms, depending on the contingencies of the population making the judgment.

Of course, it does not follow from empirical data which shows that people *perceive* the relation between cognitive and moral capacities to be a certain way that their perception is correct. But this empirical statement does not undermine the argument that we make. Even if scientific understanding of the relation between cognitive and moral capacities is lacking, it does not change the fact that persons are moral agents embedded in a moral community, within which norms of mutually beneficial interaction must be agreed and established.

The essential point to be emphasized here is that moral thought, feeling, judgement, and behavior all occur at, and are only fully intelligible at, the level of the experiencing person, and not at the level of the brain or a particular sense organ, biological, or neurological system alone. It is *persons* within a moral community who make judgements about the moral status of the actions of others irrespective of the biological and neurological separability or otherwise of the functions that give rise to the actions being taken. We should be clear here that it is not that we should stop trying to understand the neurobiological basis of capacities such as, for example, memory, since no doubt it is important to understand *how* the components of such a capacity operates. However, understanding how such a capacity operates necessarily involves recognizing that it is realized in a *person* doing the remembering since it is part of the nature of a memory that *somebody* recalls it. The “whole person” perspective for understanding even a putatively “cognitive” capacity such as memory is indispensable because if we reflect on what it is to have a particular memory of a particular event in the past, we experience something both cognitive and affective that is neither amenable to separation into these as theoretically distinct components nor identical to them in view of the fact that these components of memory are integrated into a unified first person experience.

By extension, although the specificity available from studying the scientific basis of certain capacities via experiments is a vital component in understanding those capacities, it is insufficient for a complete account. A complete account can only be achieved by considering how those capacities are embodied and the role that they play in a particular individual’s life. It is not that trying to identify the relation between these components is unimportant; rather it reminds us that: (i) the level of explanation that we employ when thinking in normative terms is that of the acting person; and (ii) this level of explanation is therefore constitutive of the norms by which judgements are made. A capacity such as memory should therefore be understood as a hybrid, containing evaluative as well as descriptive components. We suggest that this lends support to our argument that the cognitive and moral capabilities cannot be separated in the neat way that theoretical assumptions might suggest.

To demonstrate why in some more detail we will use an approach most stridently advanced by Peter Hacker (2004, 2012) in relation to confusions in descriptive language pertaining to mind and brain in neuroscience and neuropsychological research. We argue that to describe action and functioning at the level of a putative distinction between cognitive and moral capacities is to mistakenly ascribe capacities which, although apparently distinct, can only be comprehensively accounted for at the level of the whole person. Hacker’s primary critique of the language of neuroscience, cognitive science, and psychology is that they employ a conceptual schema that is inadequate for representing the phenomena to be explained. This critique holds that terms are used which, although apparently appropriate, are misleading and in attempting to make sharp distinctions between cognitive and moral capacities misrepresent what can intelligibly describe human activity. Smit and Hacker (2014, p. 1087) state that:

We see human behavior, nonverbal, and linguistic behavior, as informed by…thought, feeling, purpose and intention, in the context of complex social conventions. That we so see is not a matter of inference, but an aspect of the human form of life.

If this is correct, it is a mistake to categorize cognitive and affective states as ontologically discrete. Rather, what can be described is what a person does or what results from a person thinking, feeling, deciding, acting, and so on. Following this line of argument, the error in the debate concerning cognitive and moral enhancement is in conceiving the two as fundamentally different kinds of capacities which can be modulated or upregulated independently to achieve particular kinds of outcomes. If this were the case then it would be possible, for example, to cleave cognitive responses from emotional ones. When we reflect on this, however, as Hacker (2004, p. 204) points out, we see that it is not possible:

Emotions are linked in complex ways to what the agent knows or believes. For in so far as an emotion must have a proper object in order to qualify as the emotion it is, the agent must take the object of his emotion to satisfy the formal characteristics which determine the object as appropriate. If he fears A or A’s action, he must believe that A and A’s action are a threat. If he feels pity for another, he must believe that person to have suffered a misfortune. If he feels regret, remorse or guilt, he must believe that he has done something unfortunate or wrong…If one fears A, it is because one knows or believes that A threatens an interest one has. One will normally have reasons for thinking that this is so, reasons one may adduce to explain or justify one’s fear. Hence one’s emotions can be reasonable or unreasonable.

According to this account, it is the *person* who must think, feel, decide, and act in a particular set of contingent circumstances, and it is only by considering this totality that we can gain insight into the nature of ethical thought and behavior. Given that thinking and feeling are both aspects of the mental, reflection on *what it is like* to be a person with agency underlines the interconnectedness of cognition and emotion that is involved in moral decision-making. Although “the mind” is the seat of these activities, it is not a physical entity but a form of shorthand or *a way of describing what it is possible for humans to do*. Given that the mind is not a physical object but a conceptual one, it is only valuable to posit distinctions within it between different kinds of mental events to a limited and commensurately conceptual extent.

It is undoubtedly the case that different regions of the brain and the manipulation thereof are causally implicated in certain kinds of mental states to varying degrees of predictability. However, since no scientific investigation will reveal “the mind” as an object of empirical study, once one moves from the conceptual to the physical, the putative distinctions made when talking at the level of the former are inadequate when attempting to map them on to the latter. As Hacker (2012, p. 12) states:

…psychological attributes are attributes of an animal as a whole…It is not the mind that is in pain, has a stomach-ache or sore-throat, but the human being. The mind cannot be characterized in terms of its thinking and being conscious, since it is the human being who thinks and is conscious…it is the human being, the person, who has a body; and also has a mind. But to have a mind, and to have a body, is not to stand in a relation to anything – it is to have and to exercise a range of powers and to have an array of somatic attributes.

This emphasizes what was salient in the initial quote from Smit and Hacker, namely that discussions of moral behavior cannot be neatly atomized into the different kinds of processes – affective, cognitive, behavioral, and so on – that are integrated into it. Furthermore, attempting to capture everything that is essential about one’s mental state within a picture that is reduced to one or other of these is not possible. To adopt one form of explanation over the other excludes aspects that we recognize as descriptively important for capturing the nature of first-person experience. As Harré (2013, p. 608) writes:

We find “depression” and “serotonin reuptake inhibitor” in the same sentence in a description of the state of the very same human being. Yet not so long ago, but before the rise of synaptic chemistry, people distinguished between candida bilis and atra bilis, as distinctive ways of being gloomy with respect to the cultural patterns of the day. So far as I know this distinction has not lived on into neurochemistry. So “depression” as a feature of how some people feel is a more complex human affair than a matter of correlations with reactions among the molecules of synaptic chemistry.

Taking this into account the holistic, socially, and culturally contextual nature of situations in which feelings, judgements, and actions occur must be placed in the foreground. It is only by not excluding these dimensions that a comprehensive conception of cognitive and moral capacities be achieved. Applying this characterization helps to demonstrate where thinking is erroneous in the debate about the enhancement of these capacities and their relation to normativity.

It has been recognized in the literature that much disagreement about the possibility of moral enhancement and means for achieving it follows from more fundamental disagreements about the nature of morality itself (Beck, 2015; Carter and Gordon, 2015), for example, whether it is foundationally more a matter of reason or emotion. Moreover, those defending the foundational role of emotional “non-cognitive” components of moral thought and behavior employ what appears to be a paradigmatically “cognitive” skill, namely the ability to form a rational argument which purports to show that we “ought” to adopt one position rather than another, to advance their view. The writers advancing these various arguments in turn frequently take into account the contingent circumstances in which a particular decision ought to be made and the reasons for doing so. As such it is interesting that it has been widely acknowledged in the debate that it is difficult to say with certainty what constitutes moral enhancement and how one might achieve it, and yet disagreement about this continues to go back and forth as much as it does.

We suggest that this impasse follows from how the debate about cognitive and moral capacities and the prospect of their enhancement has been framed. That neither polar account appears to have been satisfactory so far may indicate that we may have misunderstood what we are trying to describe and appraise. In view of the preceding analysis, therefore, we recommend a way forward based on the kind of analysis advanced by Hacker and others, and the need for *bioethicists* to consider the whole person rather than focus only on discrete evaluative or morally neutral component parts. Bioethics is not, after all, experimental psychology, neuroscience or pure moral philosophy; and in its empirical forms, bioethics should not be confused about its ultimately humanistic (in this case) object of interest: how life goes *for persons*.

We argue that this should be our starting point and, as we stated earlier, considering how life goes for embodied persons taken as a *condition of the ethical debate*, rather than an insufficiently fine-grained scientific obstacle that must be surmounted for the purpose of advancing our understanding. Adopting this approach reflects our experience of agency constituted by the having of feelings, thoughts, intentions, desires, abilities, and so on, existing within a particular lifeworld in which these must be deployed according to particular ends toward which ourselves and other individuals may have conflicting attitudes and beliefs. This picture, driven by our own experience, helps to show that clear theoretical distinctions between cognitive and moral capacities, and by extension their enhancement, do not obtain at the level of the embodied individual thinking, feeling, deciding, and acting in a particular social context.

## Conclusion

Although disagreement in the bioethical literature is widespread there appears to be broadly shared recognition that the ethical status of cognitive and moral enhancement is context-dependent. Despite other disagreements, bioethicists on all sides take the view that it is not possible to generalize about the permissibility or wisdom of cognitive or moral enhancement without reference to the circumstances in which it is to be done. Disagreement therefore arises from the normative weight given to various countervailing factors in situations where enhancements might be used and predictions about the likely outcome of doing so. Under analysis, disagreement about the moral status of both types of enhancement shows itself to be driven by and answerable to deeper underlying differences in moral and political outlooks.

What we find interesting, and what we have emphasized here, is that rather than viewing the apparent lack of conclusiveness in the debate so far as an impasse or an obstacle to our understanding, we should view the complexity which gives rise to this as *a condition* of it, and take the view that “it’s complicated” as a rational assessment of the state of our current knowledge and the basis on which to proceed. It is indeed true that normative judgements abstracted from a particular context in applied ethics make no sense, since ethics is necessarily other-considering. Indeed, that this statement implies both rationality and empathy underwrites the validity of our claim that cognitive and moral capacities are not separable in the way that the putative theoretical distinction between them suggests. One does not need to be a moral relativist to recognize that norms differ and as such that disagreement about morality is inevitable, such that, for example, certain cognitive abilities may happen to be particularly valued in particular societal groups and not others, according to the particular norms that obtain. Moral and cognitive capacities cannot be neatly disaggregated into discrete evaluative and morally neutral components since what is salient about the way we deploy this set of capacities is much broader than this.

Decisions and actions of individuals are necessarily contextual since none occur in abstraction from an embodied agent bearing a particular set of relations to others. In view of this, they are subject to competing evaluative judgements informed by their context and the other individuals on whom those decisions and actions have an impact. As we have seen, even putatively morally neutral capacities have normative significance depending on the context in which they are deployed. In this respect, it is mistaken to make a strict “real-world” division between capacities (and by extension the decisions and actions that follow from them) as strictly cognitive or moral in nature, driven by delineated biological or neurological subsystems of the person in whom they reside and in isolation from each other. To do this is to commit a mereological fallacy, namely to attribute to a part of something that can only be attributed to the whole. This misattribution at the conceptual level infects the structure of the debate which is built upon it. Therefore, as we have argued, taking the whole person, rather than interdependent component parts thereof, within a particular social context as the unit of explanation for thinking, feeling, judging, perceiving, remembering, deciding, and acting will allow for a richer and more thorough discussion of the cognitive and moral dimensions of human (bio) enhancement.

## Author Contributions

GP and AM conceived the research, carried out theoretical analysis, and wrote the paper. IS contributed to the analysis and critically reviewed the paper.

## Conflict of Interest Statement

The authors declare that the research was conducted in the absence of any commercial or financial relationships that could be construed as a potential conflict of interest.

## Footnotes

1 Regrettably in the interests of space we must sidestep the issue of what or who would count as a “morally normal agent,” given pervasive philosophical disagreement about the nature of morality and morals, on the subject of which there is an inexhaustible literature. Nevertheless, it is important to note the significance of the issue and give at least a minimal account of what we might mean. Irrespective of which competing account one happens to agree with, being “morally norm” may be understood either in the descriptive sense of conforming to the norms of a given social context, or in the normative sense of acting in ways which all rational persons ought to act.

2 The philosophical literature about the relation between reason and emotions and the relative importance of their roles in moral understanding, reasoning, judgement, and behavior is vast, and there is insufficient space to do justice to this within the context of this paper. Competing conceptions of these relationships date back as far as Plato and Aristotle, they have been the focus of investigation for major philosophers such as Locke and Hume, and debate is still live with respect to these questions in contemporary philosophy, psychology, neuroscience, biology, and so on. As such, we cannot expand further on Jotterand and Levin’s attribution of a “false dichotomy” between reason and emotions; however, we suggest that the apparent and persistent insolubility of the debate may give us grounds for advancing the kind of argument that we make here which disputes the existence of a clear distinction between the two, in view of our own experience as rational moral agents.
